# Controlled Preparation of Single-Walled Carbon Nanotubes as Materials for Electronics

**DOI:** 10.1021/acscentsci.2c01038

**Published:** 2022-11-01

**Authors:** Yuguang Chen, Min Lyu, Zeyao Zhang, Feng Yang, Yan Li

**Affiliations:** † Beijing National Laboratory for Molecular Science, Key Laboratory for the Physics and Chemistry of Nanodevices, State Key Laboratory of Rare Earth Materials Chemistry and Applications, College of Chemistry and Molecular Engineering, Peking University, Beijing 100871, People’s Republic of China; ‡ Department of Chemistry, Southern University of Science and Technology, Shenzhen, Guangdong 518055, China; § PKU-HKUST ShenZhen-HongKong Institution, Shenzhen 518057, People’s Republic of China

## Abstract

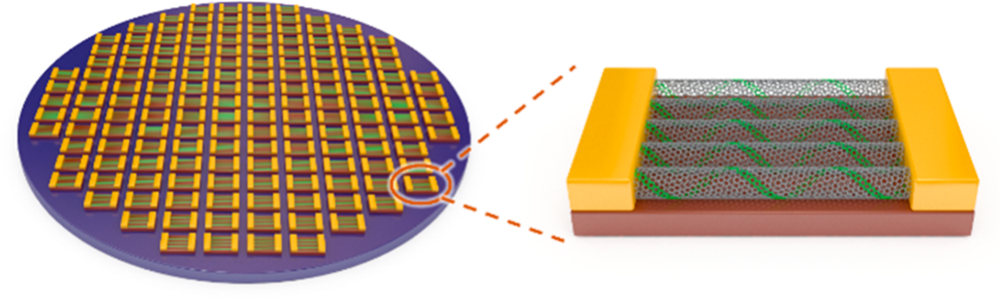

Single-walled carbon nanotubes (SWCNTs) are of particular interest as channel materials for field-effect transistors due to their unique structure and excellent properties. The controlled preparation of SWCNTs that meet the requirement of semiconducting and chiral purity, high density, and good alignment for high-performance electronics has become a key challenge in this field. In this Outlook, we outline the efforts in the preparation of SWCNTs for electronics from three main aspects, structure-controlled growth, selective sorting, and solution assembly, and discuss the remaining challenges and opportunities. We expect that this Outlook can provide some ideas for addressing the existing challenges and inspire the development of SWCNT-based high-performance electronics.

## Abstract 2

The practical application of single-walled carbon nanotubes in high-performance electronics must be based on full development of structure-controlled growth, selective sorting, and solution assembly.

## 1 Introduction

High-performance microprocessors containing very-large-scale integrated circuits (ICs) of silicon-based field-effect transistors (FETs) are the cornerstones of modern computing and communicating applications that dominate the progress of modern industry and our daily life. In order to meet the increasing demand for high performance and more complex application scenarios, researchers have been exploring new electronic materials, such as carbon nanotubes (CNTs), graphene, transition-metal dichalcogenides, and III–V semiconductors.^1−4^ Among them, CNTs are of particular interest.^5−8^

Semiconducting single-walled CNTs (s-SWCNTs) are applicable for FETs as channel materials due to their unique structure and excellent properties. The quasi-one-dimensional topology and ultrathin tube diameter of SWCNTs are beneficial to minimizing the short-channel effects and realizing superior gate control under extreme device scaling.^9−11^ The low carrier effective mass,^1^ high and symmetrical carrier mobilities (intrinsically up to 100000 cm^2^/(V s)),^12^ high current-carrying capacity,^13^ and quasi-ballistic transport^13^ of s-SWCNTs enable a high driving capability and high-speed switching at low voltages. The current density and transconductance are respectively 25 μA^13^ and 55 μS^10^ per nanotube as reported. The high thermal, chemical, and mechanical stability in carrier transport with outstanding flexibility provide devices with resistance to extreme working conditions, such as high temperature,^14^ cryogenic temperature,^15,16^ high-energy radiation,^17^ and strains.^17,18^ As one-dimensional direct-band-gap semiconductors that exhibit naturally polarized, narrow-banded, and peak-tunable light emission and absorption in the near-infrared spectral range, SWCNTs can also be applied in on-chip optical interconnects.^19−23^

**Figure 1:**
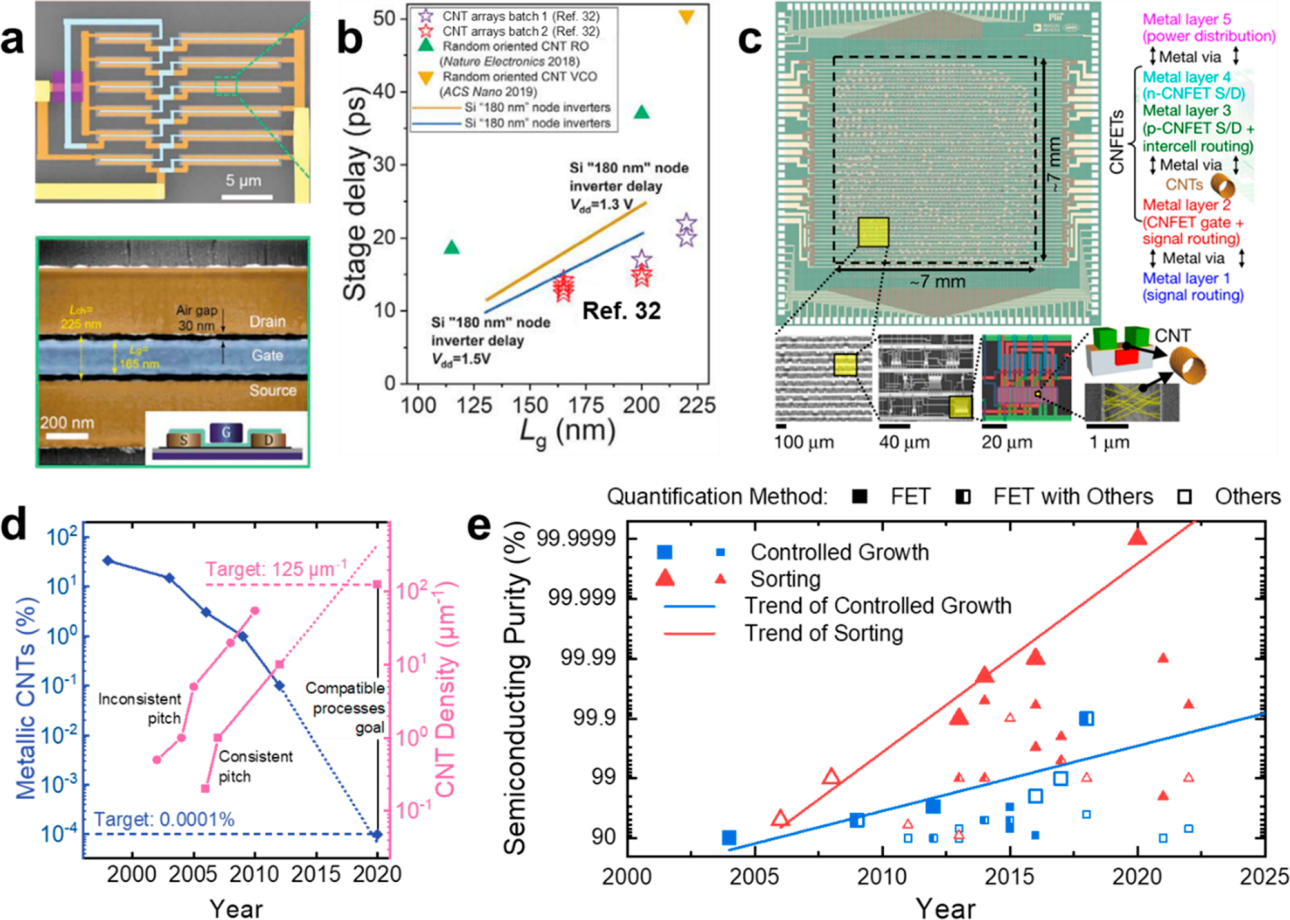
SWCNTs as materials for electronics. (a) False-colored scanning electron microscopy (SEM) images of an SWCNT-array-based ring oscillator with an inset schematic illustration of the gate structure. (b) Benchmarking of the stage delay of the champion SWCNT-array-based ring oscillators with state-of-the-art “0.18 μm” silicon devices. (a) and (b) are reproduced with permission from ref (32): copyright 2020, American Association for the Advancement of Science. (c) Image of the SWCNT-based modern microprocessor chip RV16X-NANO with SEM images of increasing magnifications exhibiting the layout of SWCNT FETs. Reproduced with permission from ref (33): copyright 2019, Springer Nature. (d) Progress on the purity and placement of SWCNTs in FETs summarized and predicted by Franklin in 2013. Reproduced with permission from ref (40): copyright 2013, Springer Nature. (e) Progress in improving the semiconducting purity of SWCNTs by controlled growth (blue squares) and sorting (red triangles). The larger icons show the milestones for fitting the trend lines.

Over the past 25 years, SWCNT FET technology has matured in the laboratory. The first p-type transistor fabricated by Dekker et al. in 1997^24^ showed a small device current because the Schottky barrier between Pt electrodes and SWCNTs hindered hole injection. With the successive exploitation of Pd electrodes^13,25^ and Sc/Y electrodes,^15,26^ which exhibit perfect Ohmic contacts with the valence and conduction bands of SWCNTs, respectively, both p-type and n-type SWCNT FETs with performance approaching the ballistic limit have been realized. On this basis, doping-free symmetrical complementary metal oxide semiconductor (CMOS) circuits,^27^ digital logic gates,^28−30^ and a computer composed of 178 SWCNT transistors^31^ were fabricated. In general, SWCNT FETs have the advantages of low power consumption and high frequency. For example, individual SWCNT-based FETs with gate lengths as short as 5 nm outperformed state-of-the-art Si FETs in supply voltage and pitch-normalized current density.^10^ SWCNT-array-based ICs exhibited a real speed higher than that of conventional Si ICs with similar gate lengths (Figure 1a,b).^32^ The feasibility of integrating SWCNT FETs has been verified by a modern microprocessor comprising more than 14000 FETs (Figure 1c).^33^ Recently, SWCNT ICs have demonstrated rich application potential in fields such as wireless communication,^34^ neuromorphic computing,^35^ wearable devices,^36,37^ and biosensing platforms.^38^

Despite these advances, some factors still severely limit the large-scale fabrication and industrialization of SWCNT FETs. The issues of material purity and array assembly are critical ones, as previously revealed by Avouris^39^ and Franklin.^40^ SWCNTs are categorized into various chiralities indexed by two integers (*n*,*m*) that determine the tube diameter and band structure.^41^ Only two-thirds of these chiralities that meet the condition of (*n* – *m*) MOD 3 ≠ 0 correspond to semiconducting species, of which the band gap is approximately inversely proportional to their diameters.^42,43^ The other one-third corresponds to metallic species. Even just one metallic SWCNT (m-SWCNT) in the channel will short-circuit the FET. In a competitive very-large-scale IC, SWCNTs are required to be of semiconducting purity >99.9999% and assemble into highly ordered monolayer arrays of high density with a consistent tube pitch of 5–10 nm (100–200 tubes/μm) (Figure 1d), to exhibit a high on/off ratio and sufficient driving ability without inefficient metal contacts and harmful intertube screening caused by poor alignment and bundling.^32,40,44^ Furthermore, to minimize the device-to-device variation caused by differences in band gaps, s-SWCNTs with a narrow diameter distribution around 1.2–1.7 nm,^45^ or better with a suitable chirality, are preferred.

The goal in the controlled preparation of SWCNTs is to control the electrical structure, which is basically the process of band-gap engineering in the semiconductor industry. The primary target is to prepare highly pure s-SWCNTs by controlled growth and sorting (Figure 1e), and the ultimate goal is to prepare s-SWCNTs with identical band gaps (determined by chiralities) in adesirable range. The highest semiconducting purity achieved to date by controlled growth is close to 99.9%,^46,47^ and the chirality purity is ∼97.4%.^46^ The highest semiconducting purity achieved by sorting is >99.9999% through a multistep treatment with conjugated polymers.^32^ Combining controlled growth and sorting techniques will be the solution to achieve both high semiconducting and chiral purity. Based on sorted dispersions of s-SWCNTs, various solution methods succeeded in realizing arrays with good alignment, but only a few achieved the density target.^32,34,48^

In this Outlook, we will outline the efforts to prepare SWCNTs as materials for electronics and discuss the remaining challenges and opportunities. In the following sections, the methodologies, main progress, and opinions regarding the further development of structure-controlled growth, selective sorting, and solution assembly of SWCNTs will be demonstrated. In the end, we will summarize the present situation and future directions in the field. We expect that this Outlook could give an idea of the package solution of SWCNT preparation, inspiring the development of high-performance electronics.

## 2 Controlled Growth of s-SWCNTs

Currently, chemical vapor deposition (CVD) is the most widely used method to synthesize SWCNTs. Band-gap control and tube alignment are the two key issues in the synthesis of SCWNTs for electronic applications. The ultimate goal is to grow s-SWCNTs with ultrahigh purity and identical band gap. Arrays of good alignment and high density are also desired. Though it is still far from the target, important progress has been achieved, lighting the future pathway.

### 2.1 Band-Gap Engineering in the Controlled Synthesis

#### 2.1.1 Selective Growth of s-SWCNTs by Etching and Twisting

**Figure 2:**
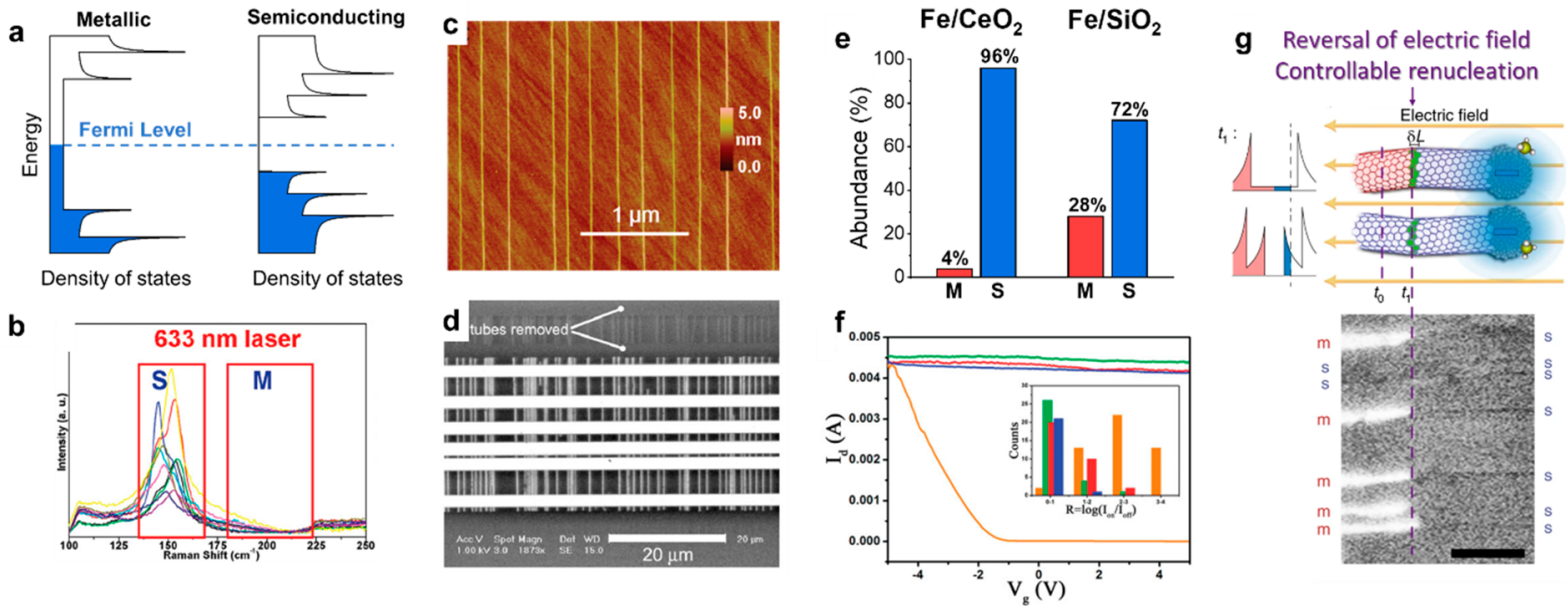
Selective growth of s-SWCNTs. (a) Schematic of density of states of m- and s-SWCNTs. (b–d) Raman spectra (b) and atomic force microscopy (AFM) image (c) of SWCNT arrays grown from a mixed feedstock of methanol and ethanol and an SEM image of the FET devices (d) fabricated with those arrays. Reproduced from ref (49): copyright 2009, American Chemical Society. (e) Statistics of m-/s-SWCNTs grown from Fe/CeO_2_ and Fe/SiO_2_ catalysts. Reproduced from ref (56): copyright 2014, American Chemical Society. (f) Typical transport characteristics of the SWCNT-based FETs. The inset shows the statistical result of on–off ratios. Blue, green, orange, red represent results of SWCNTs grown with oxygen flow rates of 0, 0.1, 0.2, and 0.3 sccm, respectively. Reproduced from ref (51): copyright 2011, American Chemical Society. (g) Upper panel: Twisting the chirality of SWCNTs by reversing the electric field to induce renucleation. Lower panel: SEM image of the SWCNTs changing from metallic (bright ones) to semiconducting (dark ones). Reproduced with permission from ref (47): copyright 2018, Springer Nature.

Since m-SWCNTs have available density of states near the Fermi level, while s-SWCNTs do not, metallic tubes exhibit lower ionization energy and higher oxidizability (Figure 2a). Taking advantage of this difference, many strategies have been developed to selectively prepare s-SWCNTs by inhibiting the growth of metallic nanotubes or etching them away.

In 2009, Liu et al. discovered that horizontally aligned arrays of s-SWCNTs were selectively grown on quartz substrates when an appropriate amount of methanol was added to the ethanol feedstock, initiating a large number of explorations with similar strategies (Figure 2b–d).^49^ The tubes showed a semiconducting selectivity of ∼95% and a narrow diameter distribution of 1.4–1.8 nm. The ^•^OH radical was believed to play the role of etchant of m-SWCNTs. Diameter confinement from the quartz substrate was recognized as an essential factor that ensured the selective etching.^50^ More etchants such as oxygen (Figure 2f),^51^ water,^52^ and isopropanol,^53^ as well as plasma^54^ and UV light,^55^ have also been adopted. However, the growth window for a decent selectivity is normally very narrow; therefore, the CVD conditions need to be strictly controlled. Nonetheless, CeO_2_-supported catalysts have been shown to be very robust in the selective growth of s-SWCNTs (Figure 2e).^56^ Due to its oxygen storage capacity, CeO_2_ can steadily maintain an oxidative environment and inhibit the growth of m-SWCNTs, guaranteeing reproducible selectivity.

An intrinsic challenge of this etching-indispensable strategy is the trade-off between selectivity and yield. High selectivity can only be achieved when the etching effect is strong with low growth efficiency. When the content of s-SWCNTs was increased from 67% (nonselective) to 98%, the yield was reduced by a factor of ∼1000 as reported.^57^ The yield could be increased through multicycle growth,^58^ but was far from satisfactory.

Jiang et al. developed a unique strategy to twist m-SWCNTs into s-SWCNTs by electro-renucleation during growth, achieving a selectivity as high as 99.9%.^47^ The differences of formation energy between s- and m-SWCNTs during growth were significantly amplified by the reversal pulse of the electric field, thereby inducing renucleation of m-SWCNTs to s-SWCNTs (Figure 2g). This strategy is suitable for the growth of horizontally aligned arrays of s-SWCNTs due to their identical growth direction parallel to the electric field.

#### 2.1.2 Selective Growth of s-SWCNTs via Chirality Control

**Figure 3:**
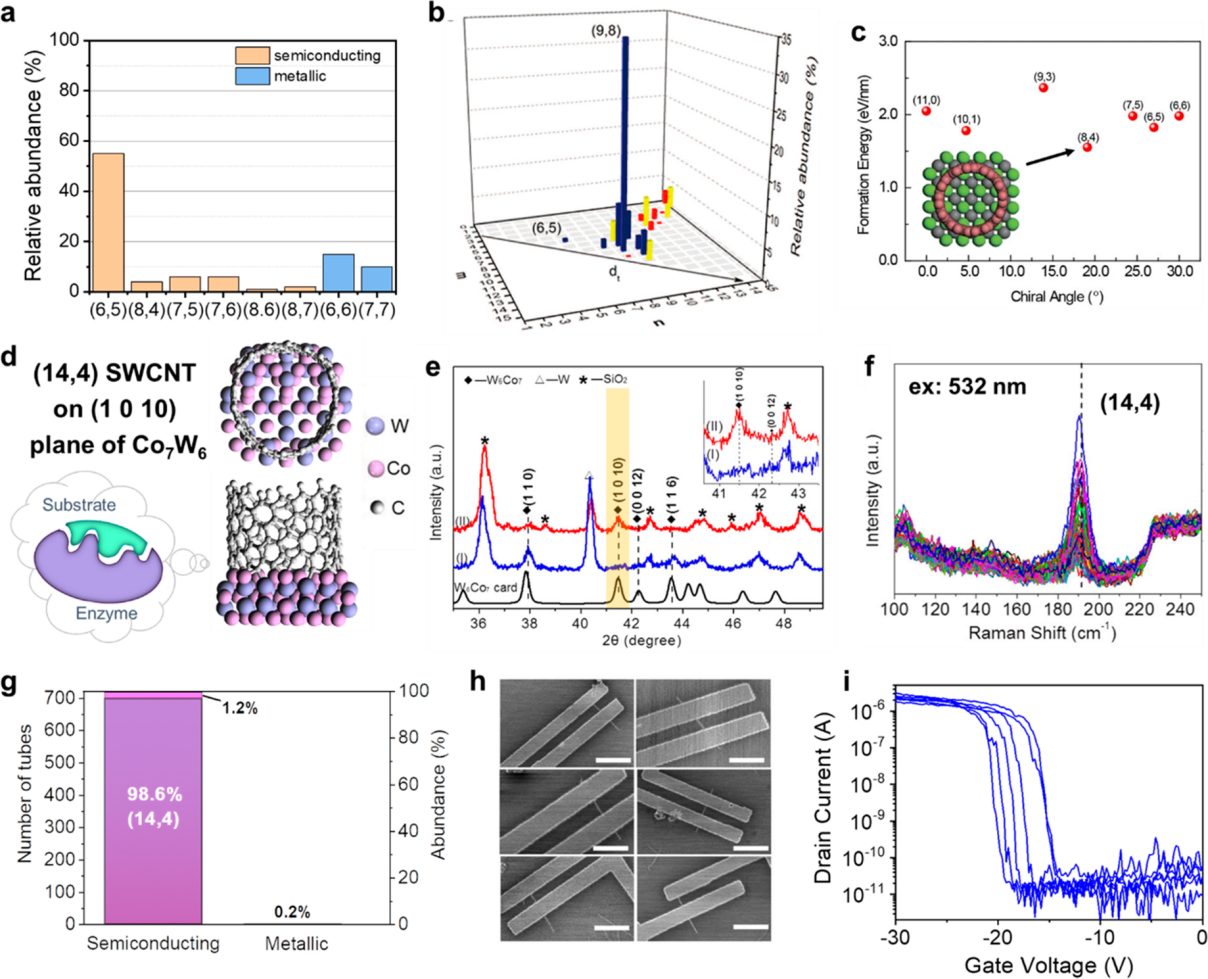
Chirality-controlled growth of s-SWCNTs. (a) Abundances of different chiralities of (6,5)-enriched SWCNTs grown from CoMo catalyst. Reproduced from ref (60): copyright 2006, American Chemical Society. (b) Abundances of different chiralities of (9,8)-enriched SWCNTs grown from the sulfate-promoted Co/SiO_2_ catalyst. Reproduced from ref (63): copyright 2013, American Chemical Society. (c) Formation energy of SWCNTs with similar diameters on a WC (100) surface (inset: simulation result showing symmetrical matching of catalyst surface with tube ends). Reproduced with permission from ref (65): copyright 2017, Springer Nature. (d) Side views and top views of the interface between a (14,4) nanotube and the (1 0 10) plane of Co_7_W_6_ from DFT simulation results. (e) XRD patterns of the catalyst reduced by pure H_2_ (curve I, blue) and by H_2_/H_2_O (curve II, red), as well as the standard card of Co_7_W_6_ (black). The peak of (1 0 10) plane is highlighted. (f) Raman spectra (RBM region) of the (14,4)-enriched SWCNTs. (g) Abundance of (14,4) nanotubes and s-SWCNTs from a statistical analysis of Raman spectra after a water vapor treatment. (h, i) SEM images (h) and transport characteristics (i) of the FET devices fabricated with the (14,4)-enriched SWCNTs. (d–i) are reproduced from ref (46): copyright 2017 American Chemical Society.

The selective growth of nanotubes with chiralities of (*n* – *m*) MOD 3 ≠ 0 also gives s-SWCNTs. In 2003, the growth of SWCNTs enriched with (6,5) and (7,5) by Resasco et al. became the first chapter of chirality-controlled growth.^59^ The selectivity was up to 55% toward (6,5) (Figure 3a).^60^ The key to selectivity lies in the design of the bimetallic CoMo catalysts, in which the Mo species disperses and stabilizes metallic Co to form small and uniform nanoparticles. A similar strategy was extended to a variety of catalysts, which exhibited selectivity toward (6,5), (7,5), or (7,6).^61,62^ Notably, Chen et al. used a sulfur-promoted Co/SiO_2_ catalyst to selectively grow (9,8) nanotubes with an abundance of 33.5% (Figure 3b).^63^ The diameter of (9,8) tube is 1.17 nm, which is larger than those of the aforementioned SWCNTs (0.75–0.83 nm) and more in line with the requirement of FET devices. They proposed that the involvement of Co_9_S_8_ intermediates benefits the formation of uniform Co nanoparticles for selective growth.

In addition to the catalysts, the chirality-dependent difference in growth kinetics may also take a role in chirality selectivity. Yakobson et al. have theoretically interpreted the kinetic favorability of near-armchair ((*n*,*n*–1) or (*n*,*n*–2)) and (2*m*,*m*) chiralities.^64^ This means when the size distribution of the catalyst is restricted to a narrow range, it is possible to achieve enrichment of a specific (*n*,*n*–1), (*n*,*n*–2), or (2*m*,*m*) chirality under suitable CVD conditions. Because tubes of (*n*,*n*–1) or (*n*,*n*–2) chiralities are always semiconducting, their advantage in kinetics brings about great convenience in the selective growth of s-SWCNTs, which was validated by the selective growth of the aforementioned (6,5), (7,5) (7,6), and (9,8) tubes.^61,62^ The enrichment of (2*m*,*m*) nanotubes was reported experimentally,^65,66^ particularly semiconducting (8,4) tubes.^67,68^ Zhang et al. explained that the enrichment of specific (2*m*,*m*) nanotubes (up to 80% for (8,4)) came from the coeffect of symmetrical matching of the catalyst surface with tube ends and their advantageous growth kinetics (Figure 3c).^65^

Inspired by enzyme-catalyzed reactions, Li et al. designed intermetallic Co_7_W_6_ catalysts with high melting point and unique crystal structure of lower symmetry than normal metallic catalysts.^69,70^ Using such catalysts as epitaxial templates combined with optimization of kinetic growth conditions, semiconducting (14,4) tubes were selectively synthesized (Figure 3d–i).^46^ The content of s-SWCNTs was 98.9%, among which 97.4% are (14,4) tubes. The purity was further improved to 99.8% for s-SWCNTs and 98.6% for (14,4) tubes by post-treatment of water vapor. The kinetically unfavorable (16,0) tubes were also synthesized at an abundance of nearly 80%.^71^ This strategy has also been demonstrated with various catalyst precursors^72,73^ and expanded to other intermetallic compounds.^74^ The strategy of combining thermodynamic preponderance (using catalysts with unique atomic arrangements as structure templates) and kinetic control (manipulating growth conditions) has been shown to be powerful in synthesizing chirality-specific SWCNTs,^41^ holding great potential in preparing s-SWCNTs with high purity.

In the studies of chirality-specified growth of SWCNTs, the identification and quantification of tube chiralities and contents are also important and challenging. Li et al. developed some feasible methods relying on both spectroscopic and microscopic techniques.^41,69,70^ Raman, Rayleigh scattering, polarized optical absorption, and selected area electron diffraction working together can give precise assignments to the chiralities of the tubes. Raman statistics and Raman combined with microscopic techniques, including AFM and SEM, can give reliable quantification of the contents of each chiralities.

### 2.2 Controlled Growth of Horizontally Aligned SWCNT Arrays

The alignment of nanotubes is generally achieved by introducing some external guiding force. Gas-flow-guided growth and substrate-lattice-guided growth are the two main strategies.

**Figure 4:**
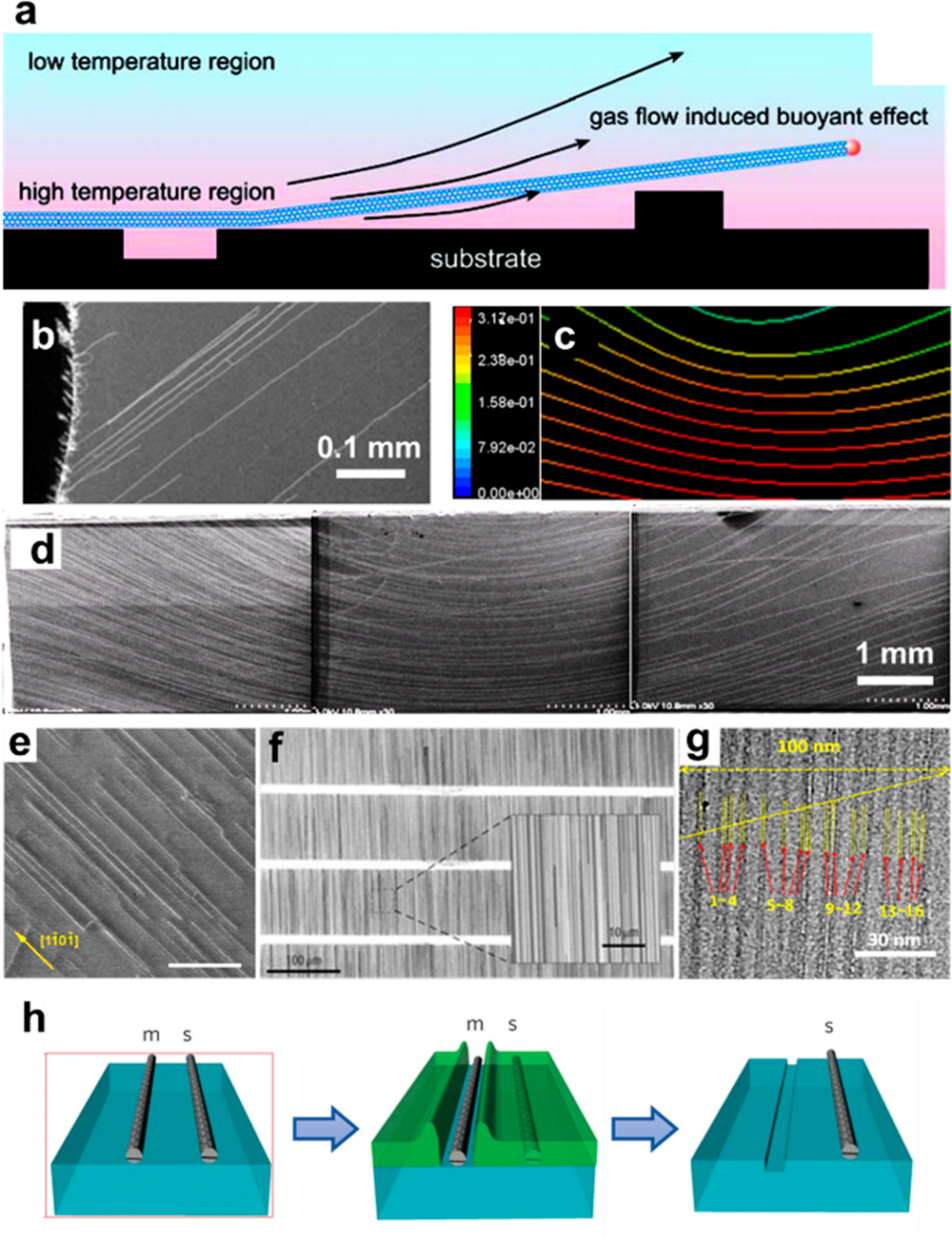
Controlled growth of horizontally aligned SWCNT arrays. (a) Schematic of the “kite mechanism” for gas-flow-guided growth. Reproduced from ref (76): copyright 2007 American Chemical Society. (b) SEM image of the gas-flow-guided SWCNTs. Reproduced from ref (75): copyright 2004 American Chemical Society. (c, d) Hydromechanical simulation of streamline (c) and SEM image of the SWCNT arrays (d) when a cylindrical barrier was placed on the side of the substrate. Reproduced with permission from ref (78): copyright 2009 IOP Publishing. (e) SEM image of an aligned SWCNT array grown on sapphire substrate. Reproduced from ref (86): copyright 2008 American Chemical Society. (f) SEM image of aligned SWCNT arrays grown on a quartz substrate with patterned catalyst stripes. Reproduced with permission from ref (81): copyright 2007, Springer Nature. (g) Transmission electron microscopy (TEM) image of an SWCNT array with a density up to 160 tubes/μm. Reproduced with permission from ref (87): copyright 2015, Springer Nature. (h) Schematic of the thermocapillary purification process, where a thermocapillary resist is first deposited on the SWCNT array, then the m-SWCNTs are selectively exposed using Joule heat, and finally the m-SWCNTs are etched away and the thermocapillary resist is removed. Reproduced with permission from ref (84): copyright 2013, Springer Nature.

Gas-flow-guided alignment is based on the so-called “kite mechanism”. A catalyst nanoparticle (together with a nanotube) floats in a gas flow above the substrate due to thermal buoyancy, and the orientation of the nanotube is thus guided by the direction of gas flow (Figure 4a,b).^75,76^ With this method, the aligned SWCNTs reached a record length of 18.5 cm.^77^ The orientation and shape of SWCNT arrays can be controlled by manipulating the flow field (Figure 4c,d).^78^ The challenge here is increasing the density, because floating nanotubes easily form bundles when the density is high. In addition, few-walled CNTs are sometimes grown, which is undesirable for device applications.

Substrate-lattice-guided alignment is based on the strong interaction between single-crystal substrates and SWCNTs. In 2005, the aligning effects of sapphire^79^ and quartz^80^ were discovered (Figure 4e,f). SWCNT arrays of excellent alignment were prepared with more than 99.9% of nanotubes lying within 0.01°.^81^ It is generally accepted that few-walled CNTs will not grow with this method. Moreover, using the “Trojan” catalyst, Zhang et al. obtained SWCNT arrays of ultrahigh density (∼160 /μm) on sapphire substrates (Figure 4g).

In the growth of s-SWCNT arrays, there is always a trade-off between purity and density. For high-density SWCNT arrays (≥100 tubes/μm), the highest semiconducting purity reported is 91%,^82^ while for SWCNT arrays of high semiconducting purity (99.9%), the highest density is ∼11 tubes/μm.^47^ Some post-etching methods have been developed to further increase the semiconducting purity of SWCNT arrays. Selective electrical breakdown by Joule heating is one of the most widely used methods, breaking down the m-SWCNTs while preserving s-SWCNTs by turning off via gate voltage.^83^ In fact, the s-SWCNT arrays used in the CNT computer reported in 2013 was prepared by this method.^31^ In order to enhance the removal of m-SWCNTs, Rogers et al. introduced a thermocapillary resist film above the SWCNT arrays (Figure 4i).^84^ All m-SWCNTs were exposed by Joule heating and then easily etched away by reactive ions. However, due to the nature of the thermocapillary flow, the spatial resolution of this method is limited to ∼100 nm. Maruyama et al. improved the spatial resolution to ∼55 nm by utilizing the exothermic oxidation of the organic films.^85^ Nonetheless, it is still challenging to apply for ultradense SWCNT arrays. Moreover, the postetching inevitably leads to an increase in non-uniformity of the local SWCNT density, thereby increasing the performance variability of FETs and weakening its usability in large-scale ICs.^6^

### 2.3 Summary of Controlled Growth of s-SWCNTs

The selective growth of s-SWCNTs by using etching agents to preferentially suppress the growth of m-SWCNTs has been widely demonstrated, but it is challenging to reach a high selectivity. However, the strategy based on electro-renucleation showed great potential in growing s-SWCNT arrays of high purity (99.9%).^47^ For chirality-controlled growth through the synergy of using the unique intermetallic Co_7_W_6_ catalyst and kinetic control, s-SWCNTs of high purity (98.9%) with 97.4% (14,4) species were synthesized.^46^ This method offers a better uniformity of band gap.

We expect that combining the electro-renucleation with catalyst design may result in much improved selectivity, which is well worth further exploration. A postgrowth treatment can further increase the purity of s-SWCNTs, though the conditions need to be finely tuned to balance purity and yield. Thus, an ultrahigh selectivity toward s-SWCNTs can be expected. However, taking the requirement of density into account, enormous efforts are still needed to establish feasible approaches to prepare s-SWCNTs for high-performance electronics.

## 3 Sorting of s-SWCNTs

Although the discovery of SWCNTs occurred in 1993,^88^ the separation of SWCNTs was not reported until this century. In recent years, the development of SWCNT sorting made the application of semiconducting or even single-chirality SWCNTs promising. Four of the dominating methods used to separate SWCNTs are density gradient ultracentrifugation (DGU), chromatography, selective extraction by conjugated polymers (SECP), and aqueous two-phase extraction (ATPE).

### 3.1 Selective Extraction by Conjugated Polymers in Organic Phase

**Figure 5:**
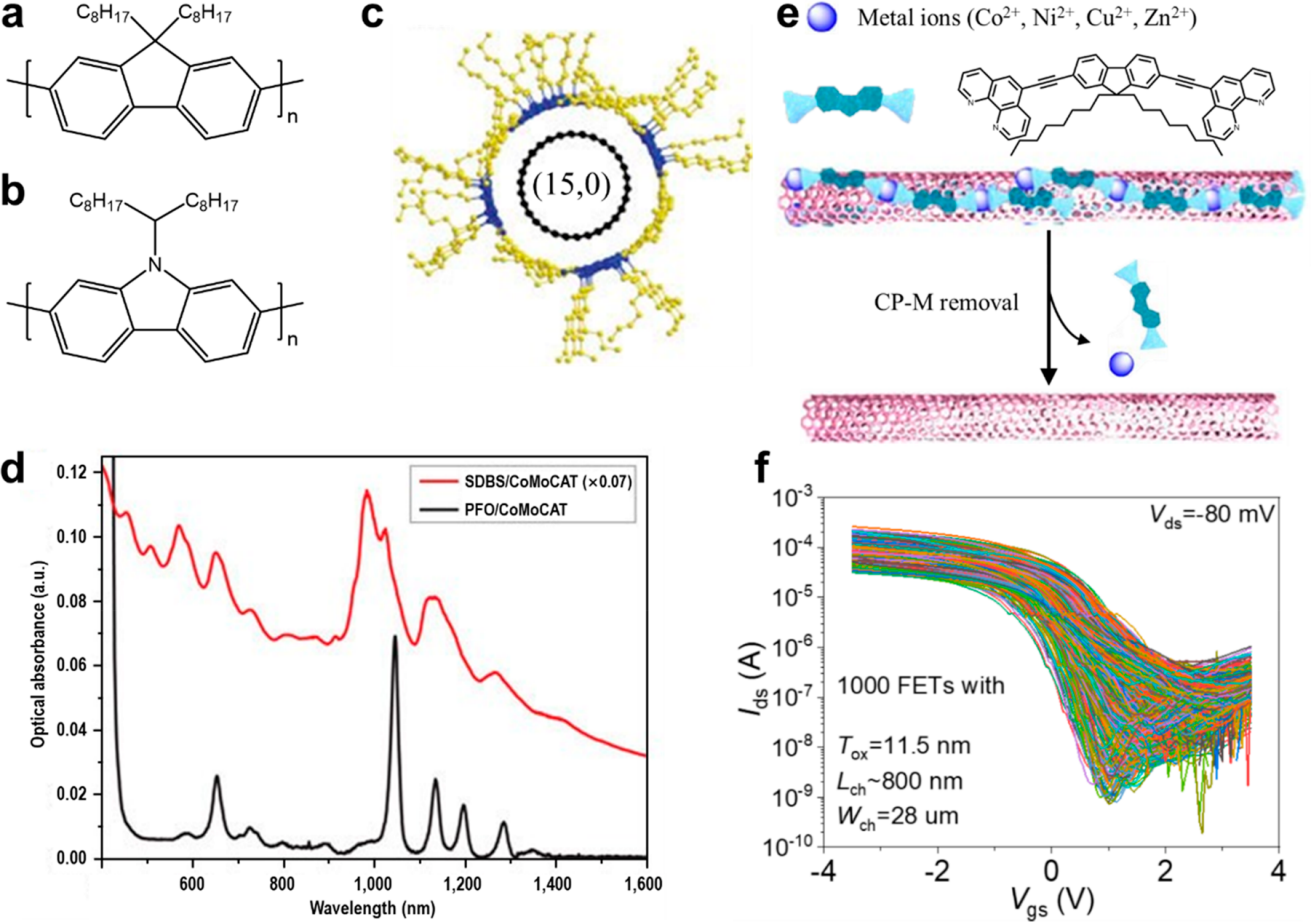
SWCNT sorting by SECP. (a, b) Molecular structure of PFO (a) and poly[9-(1-octylonoyl)-9*H*-carbazole-2,7-diyl] (PCz, b). (c) Radial view of molecular mechanics simulations of the wrapping conformation of a (15,0) PFO chain encased with six repeat units. (d) Absorbance spectra of CoMoCAT SWCNTs dispersed by SDBS in aqueous solution and PFO in toluene. (c) and (d) are reproduced with permission from ref (89): copyright 2007, Spinger Nature. (e) Schematic illustration of the solubilization of SWCNTs by coordination polymers composed of fluorene moieties and metal complexes (CP-M) and the removal of CP-M from SWCNT surface. Reproduced with permission from ref (105): copyright 2014, Spinger Nature. (f) Transfer characteristics of 1000 FETs. Reproduced with permission from ref (32): copyright 2020, American Association for the Advancement of Science.

SECP performed in an organic phase, pioneered by Nicholas et al.^89^ and Li et al.,^90^ enables the selective dispersion of s-SWCNTs in a short processing time by simple sonication and centrifugation. Conjugated polymers interact with SWCNTs through intermolecular interactions represented by π–π stacking between the conjugated units and the tube walls (Figure 5c). A possible mechanism of the selectivity toward s-SWCNTs is that the stronger polarizability of m-SWCNTs leads to stronger interactions between themselves and weaker interactions with polymers, resulting in a preferential aggregation of m-SWCNTs.^91−93^ By engineering the polymer structures, including the backbones^94−96^ and the side chains,^97,98^ the selectivity can be modified. Up to now, many kinds of conjugated polymers have been synthesized and developed for the selective extraction of s-SWCNTs, including traditional conjugated polymers such as polyfluorenes (Figure 5a), polycarbazoles (Figure 5b), polythiophenes, etc. Poly[(9,9-dioctylfluorenyl-2,7-diyl)-*alt*-co-(6,6′-(2,2′-bipyridine))] (PFO-Bpy) allows the extraction of single-chirality (6,5) tubes.^95^ In 2020, s-SWCNTs with >99.9999% semiconducting purity was achieved by repetitive sonication and filtration (Figure 5f).^32^ Compared to the sorting processes in aqueous SWCNT dispersions, the extraction pathway offers higher purity, featured by better-resolved peaks and remarkably reduced baseline in the absorption spectrum (Figure 5d), although the yield is much lower. Due to the negative effect of the residual polymer on the device performance as well as the difficulty in polymer synthesis, removable/recyclable polymers, such as degradable polymers^99−102^ and supramolecular polymers enabled by hydrogen bonding^103,104^ or coordination^105^ were also explored (Figure 5e).

### 3.2 Chromatographic Separation

**Figure 6:**
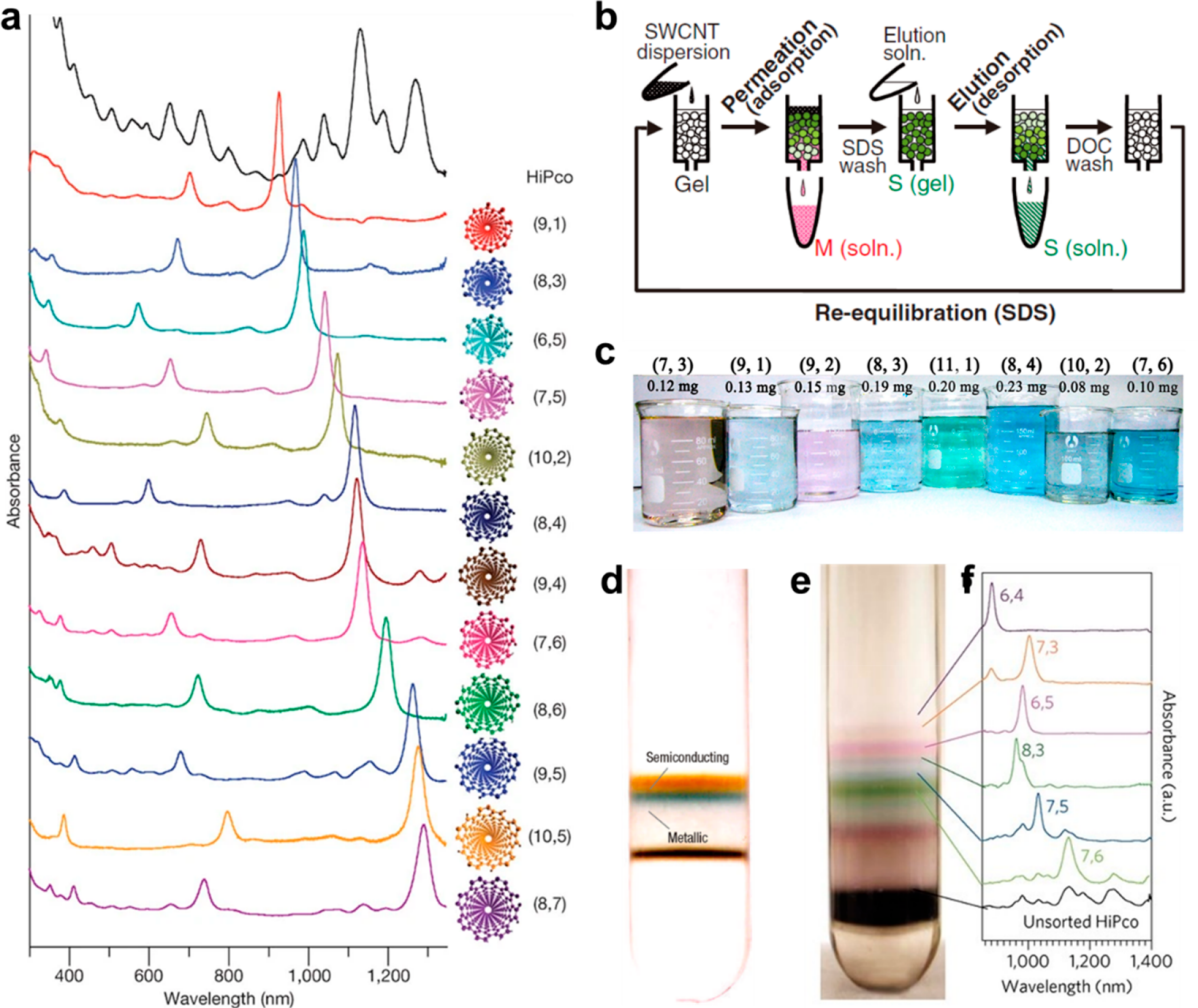
SWCNT sorting by chromatography and DGU. (a) Absorption spectra of 12 kinds of single-chirality species separated by ion exchange chromatography. Reproduced with permission from ref (108): copyright 2009, Spinger Nature. (b) Schematic of the M/S and diameter separation by gel chromatography. Reproduced with permission from ref (112): copyright 2009, The Japan Society of Applied Physics. (c) Image of single-chirality species separated on a sub-millimeter scale by gel chromatography. Reproduced with permission from ref (117): copyright 2021, American Association for the Advancement of Science. (d) Photograph of the M/S separation of surfactant-dispersed SWCNTs by DGU. Reproduced with permission from ref (120): copyright 2006, Spinger Nature. (e) Photograph of HiPco SWCNTs sorted by nonlinear DGU. (f) Absorbance spectra of the layers marked in (e). (e) and (f )are reproduced with permission from ref (126): copyright 2010, Spinger Nature.

In 2003, ion exchange chromatography was adopted by Zheng et al.^106,107^ to separate DNA-dispersed SWCNTs (DNA-SWCNTs). The specific interaction between DNA and SWCNTs resulted in the differential adsorption and retention of SWCNTs with different structures when they were eluted by a salt gradient. Single-chirality separation can be achieved by using specific recognition DNA sequences identified from the vast ssDNA library via a systematic search (Figure 6a).^108^

Gel-based SWCNT separation was developed in 2009.^109,110^ Kataura et al. remarkably enhanced the separation performance by using chromatography with columns of agarose gel or allyl dextran-based gel (Sephacryl).^111,112^ The preferable adsorption of SDS on m-SWCNTs led to the earlier elution of m-SWCNTs with weaker interaction with the gel column and the separation of m- and s-SWCNTs (Figure 6b).^112^ Similarly, the higher affinity of DOC (sodium deoxycholate) molecules to smaller-diameter s-SWCNTs was employed for diameter separation (Figure 6b).

Overloading,^111^ temperature,^113^ pH,^114,115^ and the addition of salts^114^ or ethanol^116^ could amplify the differential interaction between different (*n*,*m*) species, thus enabling high-yield and high-resolution chiral sorting (Figure 6c).^117^ In addition, the sorting can be automatically performed on commercially available chromatography equipment, which is a big advantage of this method.^118^

### 3.3 Density Gradient Ultracentrifugation

**Figure 7:**
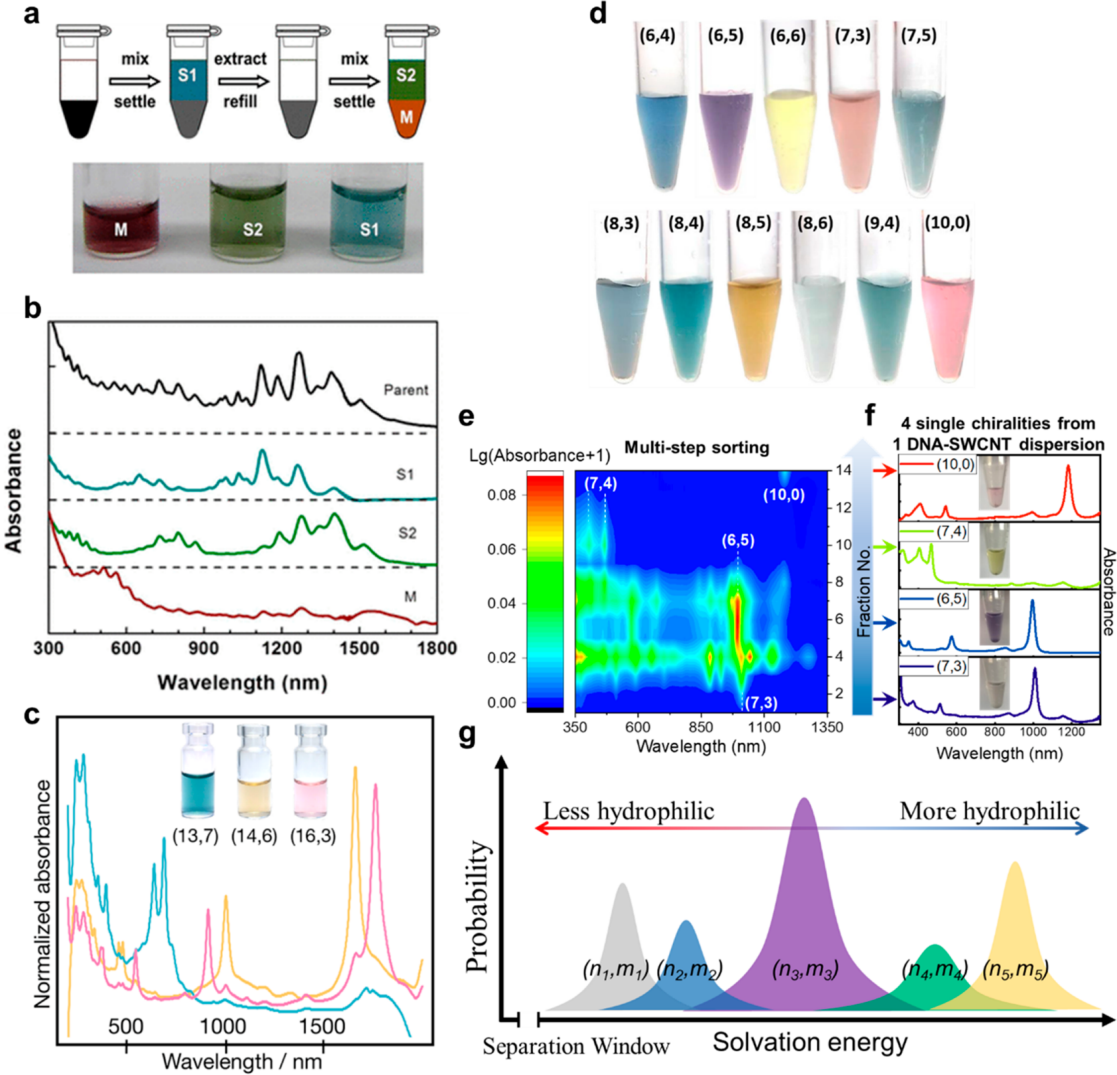
SWCNT sorting by ATPE. (a) Schematic of the oxidative extraction with sufficient oxidant added to fully oxidize SWCNTs and a photograph of the separated metallic SWCNTs and small-band-gap and large-band-gap SWCNTs. (b) Absorbance spectra of separated metallic SWCNTs and small-band-gap and large-band-gap SWCNTs. (a) and (b) are reproduced from ref (130): copyright 2015, American Chemical Society. (c) Absorption spectra of (13,7), (14,6), and (16,3) sorted by ATPE of alkane-filled SWCNTs. Reproduced from ref (132): copyright 2020, American Chemical Society. (d) Photograph of the single-chirality species sorted by ATPE of DNA-SWCNTs with the assistance of machine-learning-guided screening of DNA sequences. Reproduced from ref (134): copyright 2022, American Chemical Society. (e) Contour plot of the absorption spectra of each fraction obtained during the separation of TCT CCC TCT CCC TCT-SG65i. (f) Absorption spectra of (7,3), (6,5), (7,4), and (10,0) obtained from 2T, 6T, 10T and 13B fractions. (e) and (f) are reproduced from ref (136): Copyright 2019, American Chemical Society. (g) Schematic of the solvation energy spectrum.

DGU was introduced into SWCNT soring by Hersam et al. in 2005.^119^ Separation was achieved by the equilibrium sedimentation formed when the density of the dispersoids was the same as the density of the surrounding medium. In this system, dispersant–SWCNT hybrids are the dispersoids, whose density is determined by not only the intrinsic density of SWCNTs but also the surface coatings,^120^ counter-ions,^121,122^ hydration layers,^123^ and encapsulated species inside the tubes.^124,125^ Surfactants take important roles: for example, using sodium cholate (SC) as the dispersant alone led to diameter sorting, while using SC and SDS together led to m- and s-SWCNT (M/S) sorting (Figure 6d).^120^ By using nonlinear DGU with density gradient profile varying gently with depth, the resolution was significantly improved. Weisman et al.^126^ separated 10 fractions containing nearly single chirality species (Figure 6e,f) and 7 single-chirality enantiomers.

### 3.4 Aqueous Two-Phase Extraction

The study of ATPE for SWCNT was initiated by Zheng et al.^127^ in 2013. The difference in the hydration energies of surfactant–SWCNT complexes led to their different distributions between the top and bottom phases, sorting SWCNTs of different properties. Through adjusting the surfactant composition and concentration,^128^ salt concentration,^129^ redox condition,^130^ and pH^131^ separately or in combination, the competitive adsorptions of surfactants on SWCNTs were modulated to improve the sorting resolution. By tuning the oxidative condition, M/S-based and band-gap-based sortings were realized (Figure 7a,b).^130^ The endohedral filling of SWCNTs further improved the sorting resolution, allowing the separation of large-diameter (13,7), (14,6), (15,5), and (16,3) tubes (Figure 7c).^132^

The sequence-dependent interaction between DNA and SWCNTs enabled high-efficiency chirality sorting of SWCNTs. By carefully selecting DNA sequences, 23 single-chirality SWCNTs were isolated.^133^ Machine-learning-guided screening of DNA sequences^134,135^ greatly improved the efficiency and success rate (Figure 7d). The average molecular weights of phase-forming polymers also have a significant influence on the distribution of DNA-SWCNTs.^136^ The sorting resolution can be improved by selecting suitable polymer combinations with the right molecular weights (Figure 7e,f).

The sorting mechanism was interpreted by a solvation energy spectrum.^136,137^ Different DNA-(*n*,*m*) species in a given DNA-SWCNT dispersion present different solvation energies, enabling the distribution variation in the two phases (Figure 7g).

### 3.5 Advantages and Development Opportunities of Various Sorting Methods

As summarized in Table 1, each of the separation approaches exhibits unique advantages and also its own challenges and opportunities toward the goal of separating high-purity single-chirality s-SWCNTs in a high concentration.

**Table 1:** Comparison of the Four Methods Adopted for Sorting SWCNTs

	advantage	development opportunities
SECP	high efficiency and semiconducting purity	improve the selectivity toward s-SWCNTs with diameters >1 nm and single-chirality SWCNTs
chromatography	automatic sorting	improve the concentration of SWCNTs in the eluted fraction
DGU	high SWCNT concentration	increase the sorting efficiency and throughput
ATPE	high efficiency and SWCNT concentration	improve the separation resolution of surfactant-dispersed SWCNTs

SECP enabled the separation of s-SWCNTs with the highest semiconducting purity among the four methods. In addition, the high-efficiency and simple processing steps of SECP dramatically reduced the threshold for the application of SWCNTs in electronics. Up to now, most of the device studies used SECP-separated s-SWCNTs. However, the yield of separation and the chiral selectivity still need to be improved,^95,138^ especially in the large-diameter regime.

For sorting in aqueous solution, although the semiconducting purity of sorted SWCNTs was not as good as that of SECP, the efficiency in chirality-based sorting is very impressive. The advantage of chromatographic separation is its easiness in automation, but the concentration of SWCNTs directly obtained after separation is low. High-concentration SWCNTs can be directly obtained by DGU and ATPE. However, DGU relies on high-speed and long-term centrifugation; the throughput of a single-round separation is limited by the scale of centrifugation. For ATPE, specifically resolving DNA sequences allow high-efficiency and high-concentration sorting of SWCNTs. Nevertheless, further improving the separation resolution for surfactant-dispersed SWCNTs is urgent for expanding ATPE to the large-diameter regime, making it more compatible for separating SWCNTs for device applications.

## 4 Assembly of Well-Aligned SWCNT Arrays from Dispersions

**Figure 8:**
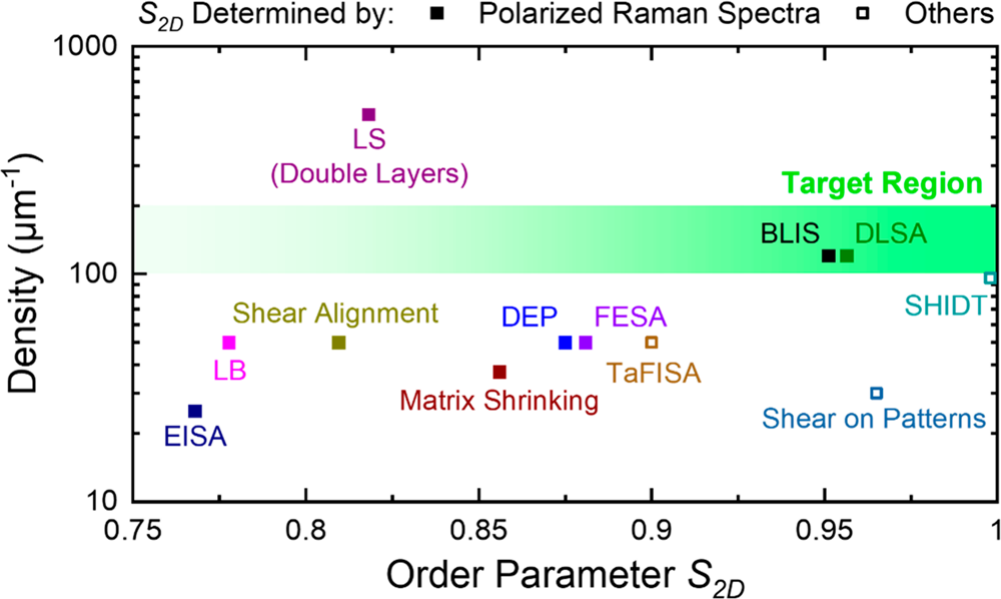
Summary of the density and order parameter *S*_2D_ of SWCNT arrays assembled by different solution methods. Abbreviations in the figure: BLIS, binary liquid interface-confined self-assembly; DEP, dielectrophoretic assembly; DLSA, dimension-limited self-alignment; EISA, evaporation-induced self-assembly; FESA, floating evaporative self-assembly; LB, Langmuir–Blodgett; LS, Langmuir–Schaefer; *S*_2D_, two-dimensional order parameter; SHIDT, spatially hindered integration based on DNA template; TaFISA, tangential flow interfacial self-assembly.

Despite a short history, the research on the alignment and assembly of SWCNTs has made rapid progress recently with the urgent need for array materials in electronics. Due to the dilemma of purity and density faced by the direct growth of SWCNT aligned arrays and the breakthrough in sorting that made it possible to obtain dispersions with extremely high semiconducting purity (up to 99.9999%), the assembly of monolayer arrays via solution processes has become the primary approach. As shown in Figure 8, various assembly methods have been developed according to different alignment mechanisms. Shear Alignment,^139^ Matrix Shrinking,^140^ and dielectrophoretic assembly (DEP)^141^ rely on anisotropic flow, stress, and electric fields, respectively. Langmuir–Blodgett (LB),^142^ Langmuir–Schaefer (LS),^143^ evaporation-induced self-assembly (EISA),^23^ floating evaporative self-assembly (FESA),^144^ tangential flow interfacial self-assembly (TaFISA),^145^ dimension-limited self-alignment (DLSA),^32^ and binary liquid interface-confined self-assembly (BLIS)^34^ utilize interfaces and contact lines to facilitate co-orientation. Spatially hindered integration based on a DNA template (SHIDT)^48^ and Shear on Patterns^146^ design the interactions between SWCNTs and patterned substrates. The arrays prepared by these methods present densities of 25–500 μm^–1^ and the two-dimensional order parameter^147,148^*S*_2D_ > 0.75.

**Figure 9:**
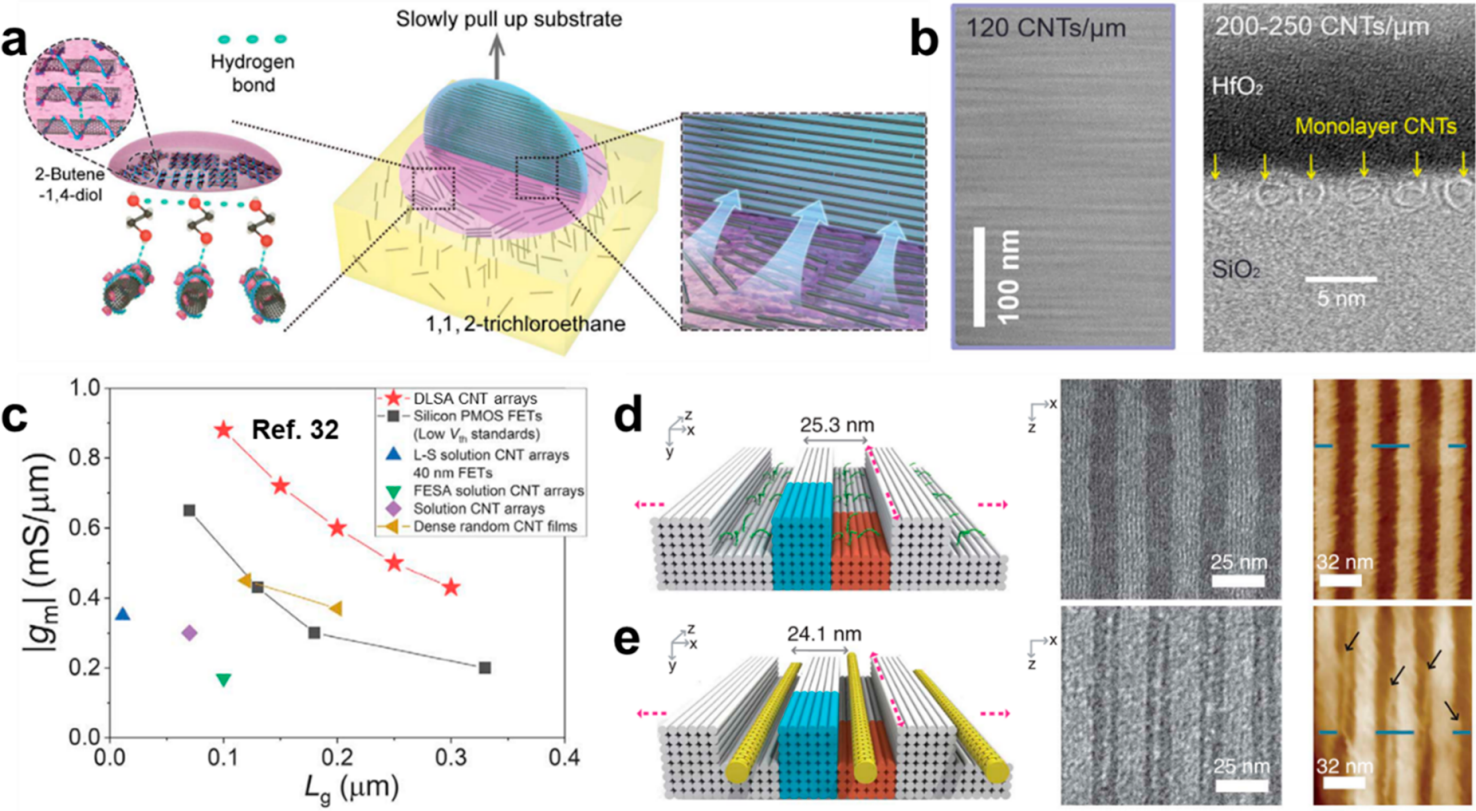
Methods for high-density assembly. (a) Schematic illustrations of the assembly process of the DLSA method. (b) SEM and cross-sectional TEM images of the assembled SWCNT arrays. (c) Benchmarking of peak transconductances versus gate lengths of the DLSA-based FETs with other reported SWCNT FETs and commercial silicon devices. (a)–(c) are reproduced with permission from ref (32): copyright 2020, American Association for the Advancement of Science. (d, e) Schematic illustrations, TEM images, and AFM images of the DNA nanotrenches before (d) and after (e) nanotube assembly in the SHIDT process. Reproduced with permission from ref (48): copyright 2020, American Association for the Advancement of Science.

Currently, few methods practically reach the density target for device applications (100–200 μm^–1^, marked in green in Figure 8). The DLSA/BLIS methods^32,34^ developed by Peng et al. achieved tube densities beyond 120 μm^–1^. It was proposed that the assembly encountered three procedures: SWCNT confinement at the liquid–liquid interface, pre-assembly, and deposition along the contact line while slowly pulling the wafer-scale substrates out of the dispersions (Figure 9a,b). Hydrogen bonding might have an important role in confining and pre-assembling SWCNTs. Top-gated FETs fabricated on these high-density SWCNT arrays showed better performance than commercial silicon FETs with similar gate lengths (Figure 9c). The SHIDT method^48^ developed by Sun, Yin, et al. used DNA origami to form nanotrenches, in which the energy advantage generated by geometric confinement and DNA hybridization promoted the selective deposition of SWCNTs (Figure 9d,e). The highest density was 96 μm^–1^, with a uniform pitch and near-perfect local alignment of nanotubes. However, the scalability of DNA origami is still challenging and the cost is very high. Cao et al. prepared bilayer arrays of SWCNTs with an ultrahigh density of 500 μm^–1^ per layer based on the LS method.^143^ Yet the FET performance was not up to expectations because of insufficient electrode contacts and severe intertube screening.

**Figure 10:**
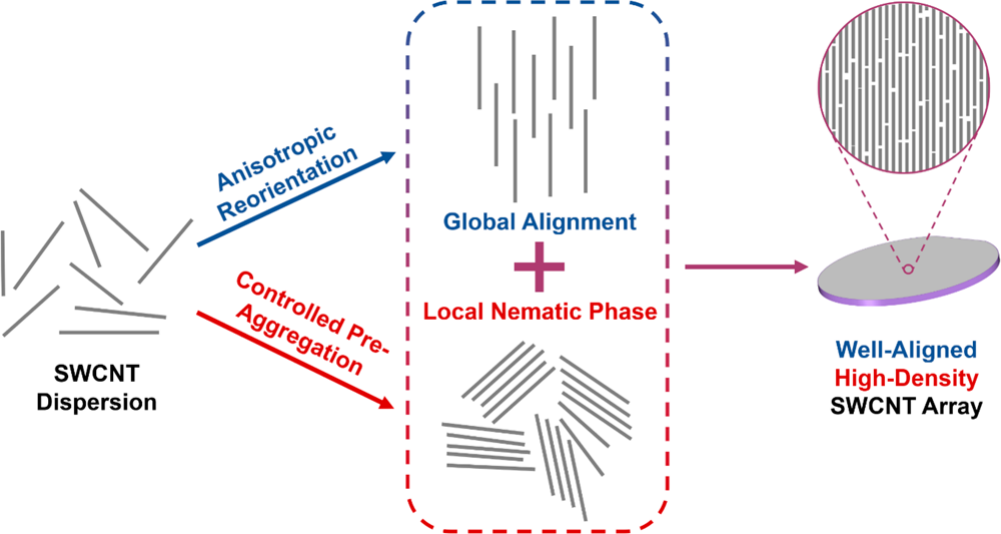
Keys to reaching good alignment and high density of SWCNT arrays.

Anisotropic reorientation and controlled pre-aggregation are two key procedures to reach good alignment and high density, respectively (Figure 10). In previous works, researchers focused more on the former. They reoriented nanotubes through physical, chemical, or topological strategies, but the densities were generally 25–50 μm^–1^, possibly due to the low tube concentration of the dispersions. Continuing to increase the concentration may lead to undesired tube bundling. In contrast, the LS method^143^ physically compressed the water surface, and the DLSA/BLIS methods^32,34^ chemically formed a potential well with hydrogen bonds. They both promoted the aggregation to raise the effective concentration of SWCNTs at the gas–liquid or liquid–liquid interface, increasing the array density to 120 μm^–1^ and above without forming large-scale bundles. We can conclude that, to increase the density of arrays, much attention should be paid to enabling the controlled pre-aggregation of SWCNTs during assembly. However, the aggregation efficiency is still unsatisfactory, which greatly prolongs the assembly time.

In addition, to further enhance the feasibility of solution assembly methods, the following challenges need to be addressed. First, the impact of interfacial fluctuations on the alignment and uniformity of SWCNTs should be optimized. The widely used FESA method^144,149^ developed by Arnold, Gopalan, et al. utilized tangential flows to reorient nanotubes along the oil–water–solid contact line. The pinning effect led to intermittent jumps rather than a continuous movement of contact lines across the pulled substrates, which created a sequential deposition of well-aligned strips and perturbed interfacial regions with random networks of nanotubes. In 2019, Rutherglen et al. significantly improved the order of nanotubes in the perturbed interfacial regions by reducing surface waves on the water subphase through isolating air flow, reducing vibration, and operating in a cleanroom.^150^ Similarly, the LS method produced arrays with better alignment than the LB method because the horizontal transfer was less disturbing to the SWCNT Langmuir film on water. On the other hand, controllably applying interfacial fluctuations to form anisotropic potential fields may also benefit the assembly of nanotubes, as revealed by early research using surface acoustic waves.^151^

Second, the effect of dispersants and solvents on the assembly process should be elucidated. The composition of SWCNT dispersions is complex and diverse. Both dispersants and solvents will affect the interactions between nanotubes and substrates, especially surfactants that significantly change the properties of surfaces and interfaces. Therefore, many assembly methods developed in specific dispersing systems have poor versatility. For example, different aqueous dispersions showed different pH ranges for deposition on poly-l-lysine-modified silicon substrates.^152,153^ The adsorption of PFO-BPy-wrapped SWCNTs on several modified silicon substrates was less favorable in toluene than in chloroform.^154^ The PCz-wrapped nanotubes in 1,1,2-trichloroethane dispersions and the poly[2-methyl-7-(6′-methyl-[2,2′-bipyridin]-6-yl)-9-(2-octylonoyl)-9*H*-carbazole] (PCO-BPy)-wrapped nanotubes in *m*-chlorotoluene dispersions were hardly deposited on silicon wafers via random adsorption, thus avoiding damage to the DLSA or BLIS process.^32,34^ The mechanisms responsible for these differences have not been fully investigated.

Third, the development of assembly methods based on aqueous dispersions should be promoted. There have been few studies on the array assembly from aqueous dispersions and no large-scale uniform features other than discrete domains,^23,141,155^ partially because of the disadvantages of aqueous dispersions such as complex composition, short nanotube length, small nanotube diameter, and low semiconducting purity. However, due to the compatibility of aqueous dispersions for the single-chirality sorting process, the assembled arrays still possess interesting performance, such as polarized light emission,^23^ which is worthy of further investigation.

## 5 Summary and Outlook

From the above demonstration and discussion, it can be concluded that the practical application of SWCNTs in high-performance electronics must be based on the full development of structure-controlled growth, selective sorting, and solution assembly steps, in which great efforts over the past 25 years have led to significant progress.

For the selective growth of s-SWCNTs, the strategy based on electro-renucleation of m-SWCNTs into s-SWCNTs showed great potential in growing aligned s-SWCNTs of high purity (99.9%).^47^ Chirality-controlled growth through the synergy of thermodynamic control using a unique intermetallic Co_7_W_6_ catalyst as an epitaxial template and kinetic control obtained both high semiconducting and chirality selectivity, resulting in materials with a better uniformity of band gap.^46^ In addition to the high selectivity, achieving alignement will be an additional advantage for application. There is still another challenge of scalable production.

For sorting, s-SWCNTs with a semiconducting purity of 99.9999% and more than 30 types of single-chirality s-SWCNTs have been separated. However, the separation of large-diameter (>1.2 nm) single-chirality s-SWCNTs is still a challenge. In the current stage, SWCNT sorting in the aqueous phase faced the common problem of short tube length and insufficient semiconducting purity. The development of a less destructive dispersing procedure and improvement of the M/S sorting resolution are crucial. As to SECP, more efforts should be made in the polymer structure engineering for higher yield and better selectivity toward larger tubes.

For array assembly, high densities of 100–200 μm^–1^ with nearly perfect alignment (*S*_2D_ ≥ 0.95) of nanotubes have been achieved with specific methods.^32,34,48^ However, low density is still the limiting factor for most methods toward practical applications, which is expected to be improved by efforts in promoting the controlled pre-aggregation of SWCNTs. Other challenges lie in optimizing surface and interfacial fluctuations to improve the uniformity of arrays, clarifying the effects of solvents and dispersants in assembly to enhance applicability for different dispersions, and developing aqueous assembly methods to expand the application of SWCNT aligned arrays with high chiral purity.

At the current stage, a critical challenge for the controlled preparation of SWCNTs is the integration of growth, sorting, and assembly. These three processes should be revisited and studied in a whole chain and optimized synergistically. The status and chirality distribution of grown SWCNTs will affect their dispersion and the efficiency of sorting as well as the utilization ratio of SWCNTs. The choice of assembly method must be based on the solvents and dispersing agents of sorted SWCNTs. The length and surface potential of sorted SWCNTs and the fluidic properties of the solution will affect the results of assembly. In addition, for the sake of reducing the variability of SWCNT FETs, which is a vital constraint of integration, more attention should be paid to the reproducibility of growth, sorting, and assembly, as well as the uniformity of the prepared arrays. SWCNT preparation should work closely with device design and fabrication, forming an entire iterative cycle. Only in this way can the long-term progress of SWCNT-based ICs be promoted.

In addition, the lack of feasible characterization methods to quantify the high semiconducting purity of SWCNTs has become a crucial constraint. For now, the only way to quantify a semiconducting purity higher than 99.9% is through fabricating FET devices with the SWCNTs and analyzing their transport characteristics, which is not only complicated but also disruptive. Moreover, an accurate analysis requires a valid determination of the length distribution and density, as well as the alignment of SWCNTs. Establishing reliable nondestructive quantification methods of good accuracy, high efficiency, and nanoscaled resolution for wafer-scale samples is a real necessity for further development of this field. We believe that the incorporation of scanning probe microscopy may shed light on a solution to this issue.

In the past 25 years, SWCNT-based electronics thrived from a single FET into a microprocessor of large-scale integration.^33^ The superior performance was demonstrated from the single FETs at extreme scaling^10^ to the level of ICs.^32^ SWCNTs have shown great potentials in both high-performance microprocessors and thin-film devices.^1^ High-quality SWCNT materials in practical availability is the prerequisite for electronic applications. We believe the future of CNT-based electronics lies in the development of SWCNT preparation.

## Abbreviations

AFM: atomic force microscopy
ATP: aqueous two phase
ATPE: aqueous two-phase extraction
BLIS: binary liquid interface-confined self-assembly
CMOS: complementary metal oxide semiconductor
CVD: chemical vapor deposition
DEP: dielectrophoretic assembly
DGU: density gradient ultracentrifugation
DLSA: dimension-limited self-alignment
DNA: deoxyribonucleic acid
DOC: sodium deoxycholate
EISA: evaporation-induced self-assembly
FESA: floating evaporative self-assembly
FET: field effect transistor
IC: integrated circuit
LB: Langmuir–Blodgett
LS: Langmuir–Schaefer
PCO-BPy: poly[2-methyl-7-(6′-methyl-[2,2′-bipyridin]-6-yl)-9-(2-octylonoyl)-9*H*-carbazole]
PCz: poly[9-(1-octylonoyl)-9*H*-carbazole-2,7-diyl]
PFO: poly(9,9-dioctylfluorenyl-2,7-diyl)
PFO-Bpy: poly(9,9-dioctylfluorenyl-2,7-diyl and bipyridine)
s-/m-SWCNT: semiconducting/metallic single-walled carbon nanotube
*S*_2D_: two-dimensional order parameter
SC: sodium cholate
SDS: sodium dodecyl sulfate
SECP: selective extraction by conjugated polymers
SEM: scanning electron microscopy
SHIDT: spatially hindered integration based on DNA template
TaFISA: tangential flow interfacial self-assembly
TEM: transmission electron microscopy
